# GLIMMERS: glioma molecular markers exploration using long-read sequencing

**DOI:** 10.1093/bioadv/vbae058

**Published:** 2024-04-15

**Authors:** Wichayapat Thongrattana, Tantip Arigul, Bhoom Suktitipat, Manop Pithukpakorn, Sith Sathornsumetee, Thidathip Wongsurawat, Piroon Jenjaroenpun

**Affiliations:** Master of Science Program in Medical Bioinformatics (International Program), Faculty of Medicine Siriraj Hospital, Mahidol University, Bangkok 10700, Thailand; Division of Medical Bioinformatics, Faculty of Medicine Siriraj Hospital, Mahidol University, Bangkok 10700, Thailand; Division of Medical Bioinformatics, Faculty of Medicine Siriraj Hospital, Mahidol University, Bangkok 10700, Thailand; Siriraj Long-read Lab (Si-LoL), Faculty of Medicine Siriraj Hospital, Mahidol University, Bangkok 10700, Thailand; Department of Biochemistry, Faculty of Medicine Siriraj Hospital, Mahidol University, Bangkok 10700, Thailand; Integrative Computational BioScience (ICBS) Center, Mahidol University, Nakon Pathom 73000, Thailand; Siriraj Genomics, Faculty of Medicine Siriraj Hospital, Mahidol University, Bangkok 10700, Thailand; Division of Medical Genetics, Department of Medicine, Faculty of Medicine Siriraj Hospital, Mahidol University, Bangkok 10700, Thailand; Department of Medicine (Neurology), Faculty of Medicine Siriraj Hospital, Mahidol University, Bangkok 10700, Thailand; Division of Medical Bioinformatics, Faculty of Medicine Siriraj Hospital, Mahidol University, Bangkok 10700, Thailand; Siriraj Long-read Lab (Si-LoL), Faculty of Medicine Siriraj Hospital, Mahidol University, Bangkok 10700, Thailand; Department of Biomedical Informatics, College of Medicine, University of Arkansas for Medical Sciences, Little Rock, AR 72205, United States; Division of Medical Bioinformatics, Faculty of Medicine Siriraj Hospital, Mahidol University, Bangkok 10700, Thailand; Siriraj Long-read Lab (Si-LoL), Faculty of Medicine Siriraj Hospital, Mahidol University, Bangkok 10700, Thailand; Department of Biomedical Informatics, College of Medicine, University of Arkansas for Medical Sciences, Little Rock, AR 72205, United States

## Abstract

**Summary:**

The revised WHO guidelines for classifying and grading brain tumors include several copy number variation (CNV) markers. The turnaround time for detecting CNVs and alterations throughout the entire genome is drastically reduced with the customized read incremental approach on the nanopore platform. However, this approach is challenging for non-bioinformaticians due to the need to use multiple software tools, extract CNV markers and interpret results, which creates barriers due to the time and specialized resources that are necessary. To address this problem and help clinicians classify and grade brain tumors, we developed GLIMMERS: glioma molecular markers exploration using long-read sequencing, an open-access tool that automatically analyzes nanopore-based CNV data and generates simplified reports.

**Availability and implementation:**

GLIMMERS is available at https://gitlab.com/silol_public/glimmers under the terms of the MIT license.

## 1 Introduction

The fifth edition of the WHO Classification of Tumors of the Central Nervous System, published in 2021, introduced significant changes in the importance of molecular diagnostics for classification and grading of gliomas (Louis *et al.* 2021). The revision brought in several well-studied markers of copy number variation (CNV) that are associated with particular classifications and grades of gliomas. For example, the complete codeletion of 1p/19q indicates oligodendrogliomas, the partial or total absence of this codeletion indicates astrocytoma, and the presence of a homozygous deletion in *CDKN2A/B* corresponds to the highest-grade glioma. However, assessment of the specified CNV markers requires sophisticated toolkits and trained technicians, and the resulting investment and costs impede practical implementation of the guidelines in clinics.

Nanopore, a long-read sequencing platform, has revolutionized the standard in sequencing machines, offering a bargain price and easy access for almost any laboratory worldwide. Several CNV analysis tools are available for use with nanopore platforms, such as ichorCNA (Adalsteinsson *et al.* 2017), SMURF (Prabakar *et al.* 2019), and Nano-GLADIATOR (Magi *et al.* 2019). While these tools are primarily designed to assess large genomic alterations, they do not specifically focus on individual CNV markers, and users unfamiliar with CNV profiling may find it challenging to interpret the results. Moreover, SMURF and Nano-GLADIATOR do not account for tumor fraction and are unable to distinguish between homozygous and hemizygous deletions at the *CDKN2A/B* locus. This distinction is critical for the accurate classification of brain tumors. It is essential to recognize that, beyond *CDKN2A/B*, an array of biomarkers exists that are instrumental in tumor classification and should be taken into account, i.e. EGFR amplification, 1p/19q codeletion, and etc. Importantly, interpreting glioma-related molecular markers, such as the co-deletion of the 1p and 19q chromosomal arms, requires a composite analysis. In this analysis, multiple CNV markers come together to conform with established diagnostic criteria. Our recent work used the nanopore platform to analyze all those CNV markers, and the results yielded high concordance with the gold standard method. (Wongsurawat *et al.* 2023) While the potential of this approach is promising, there are no bioinformatics tools currently available that are specifically designed to consolidate all markers and generate a report. This makes it challenging to reconstruct the analysis workflow for non-bioinformaticians to replicate the work.

Here, we introduce GLIMMERS (glioma molecular markers exploration using long-read sequencing) for gliomas, our new open-source tool that automates the comprehensive analysis of CNV markers for classifying and grading gliomas. This tool establishes a complete infrastructure for nanopore-based copy-number analysis and performs a range of functions including read quality control, sequence read mapping, read count normalization, CNV profiling, CNV markers extraction, and report generation. The analysis workflow is fully automated, from input handling through to the final report, enhancing both efficiency and user experience. The pipeline was developed in a cooperative Python and R environment, utilizing the subprocess module for process interaction and Conda for efficient package management. All additional package dependencies are defined in the yaml configuration file to ensure version integrity.

## 2 Methods

### 2.1 Experimental dataset

GLIMMERS was tested in our previous study (Wongsurawat *et al.* 2023) that included 3 well-characterized glioma cell lines and clinical data of 19 glioma cohort generated using the nCNV protocol (Supplementary data). The datasets can be downloaded from NCBI under BioProject ID PRJNA740254.

### 2.2 Alignment and read counting

The nanopore FASTQ file is aligned against the human genome reference (hg19 and hg38) using minmap2 v2.24; we include an additional command to eliminate secondary mapped reads with the “- -secondary=no” option (Li 2021) HMMcopy (10.18129/B9.bioc.HMMcopy) is used for read counting, GC correction, and mappability bias. (Lai *et al.* 2023) Two additional parameters, “- -window 500000” and “- -quality 20,” are added on HMMcopy.

### 2.3 Panel of normal and tumor fraction baseline

HMMcopy provides read count data in WIG file format as a read count per bin (500 000 bases). We define the deletion baseline of *CDKN2A/B* by constructing a regression model between tumor fraction and copy number value acquired from ichorCNA (https://gitlab.com/silol_public/glimmers) in the associated position. The training set is constructed by first creating an artificial admixture between a panel of normal samples and a tumor sample with homozygous deletion of the *CDKN2A/B* gene in various proportions and then checking for usability by comparing the copy number profile with the BT88 tumor cell line.

### 2.4 Marker identification, visualization, and report generation

Marker positions related to the classification of gliomas, including *CDKN2A/B*, *EGFR*, 1p/19q, and +7/−10, are identified based on the human reference genome (hg19 & hg38). After this, the corresponding bin positions that contain these identified markers are listed. An R script is then implemented for the extracted read count values, which are obtained from the read count profile (R model) derived through ichorCNA, within the specified positions. This process includes identification of the status of each individual marker. The genomic copy number profile subsequently is visualized with the ggplot2 library, so that designated bin segments are labeled and highlighted with the type of marker status. Lastly, the result is transmitted to a Python script, which generates a conclusive report.

## 3 Implementation and availability

**Figure 1.**
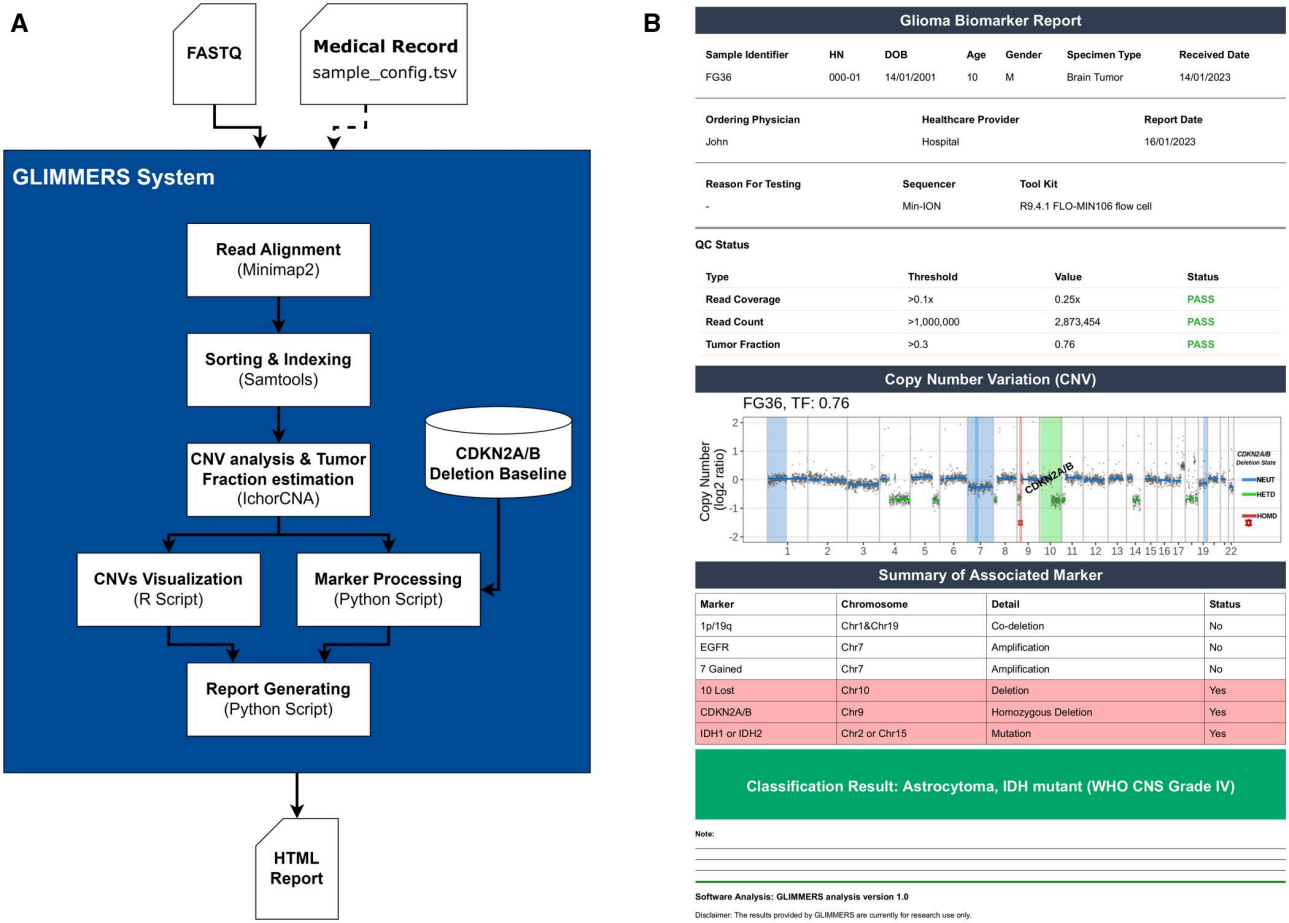
An overview workflow of GLIMMERS and example output from GLIMMERS. (**A**) The system requires a mandatory input in the form of a sequencing base, specifically in FASTQ format, along with an optional input of a medical record. Initially, the FASTQ data undergoes read alignment using Minimap2, followed by sorting and indexing with Samtools. Subsequently, the aligned reads, represented as a BAM file, progress to CNV analysis and tumor fraction estimation through IchorCNA. From the CNV results, the workflow diverges into two paths: one leading to the visualization process and the other to marker processing. The *CDKN2A/B* deletion baseline is included in predicting the deletion type of *CDKN2A/B* at this stage. Finally, the outcomes of the visualization and marker status are processed into the report-generating process, which is structured and summarized in a single report in HTML format. (**B**) The results from GLIMMERS analysis, shown in the right panel, organized into four sections for a detailed overview of the genomic profile. First, the Sample Information Header provides basic details retrieved alongside the FASTQ input, giving context to the analyzed sample. Next, the Visualization Section presents a whole genome CNVs plot with labeled segments related to glioma markers for easy identification. The status of each glioma marker is tabulated in the third section, with the alteration marker highlighted in red. Lastly, section four concludes with a summary of the GLIMMERS system’s proposed classification and grading results.

The overall system architecture of the GLIMMERS tool is depicted in Fig. 1A. The process begins with Minimap2 aligning the nanopore sequencing data (in FASTQ or compressed FASTQ [fastq.gz] file format) to the reference genome; the output data then is indexed and sorted with Samtools. Next, the CNV analysis takes place, facilitated by ichorCNA, which performs read counting within 500-kb window size and GC-bias correction with the assistance of HMMcopy. Subsequently, the data undergoes cleansing to exclude centromeres and their neighboring regions. ichorCNA uses a precomputed panel of normals and a hidden Markov model to detect copy number alterations and to calculate tumor fraction. Additionally, the tumor fraction is then used as an input to measure the deletion baseline of the *CDKN2A/B* gene. The resulting CNV data, along with the tumor fraction, are directed to the GLIMMERS data visualization and marker processing script. This script integrates positional information extracted from the CNV data, generates visualizations, and compiles the assessment of marker status. The outcomes subsequently are fed to the documentation script implemented in Python, which generates the final report that clearly details the status of each marker relevant to classifying and grading gliomas (Fig. 1B).

## 4 Conclusion

GLIMMERS is an open-source tool to facilitate assessment of CNV markers related to classification and grading of gliomas, including 1p/19q, *CDKN2A/B*, *EGFR*, and +7/−10. Moreover, this tool might detect novel deletions or amplifications at 500-kb bin; the reported work has not tested the possible limitation that GLIMMERS may detect small gene deletions or amplifications.

## Supplementary Material

vbae058_Supplementary_Data
