# Supplementary material for: GLIMMERS: glioma molecular markers exploration using long-read sequencing
Source: Bioinform Adv. 2024 Apr 15;4(1):vbae058. doi: 10.1093/bioadv/vbae058 (PMC11087932; doi:10.1093/bioadv/vbae058)
Supplement: vbae058_Supplementary_Data [file vbae058_supplementary_data.pdf]

nCNV-seq protocol

18th February 2024

S/–LoL
S/riraj - Long-read Lab

Important note: This method is specifically optimized for use with fresh-frozen samples and not recommend for formalin-fixed paraffin-embedded (FFPE) samples.

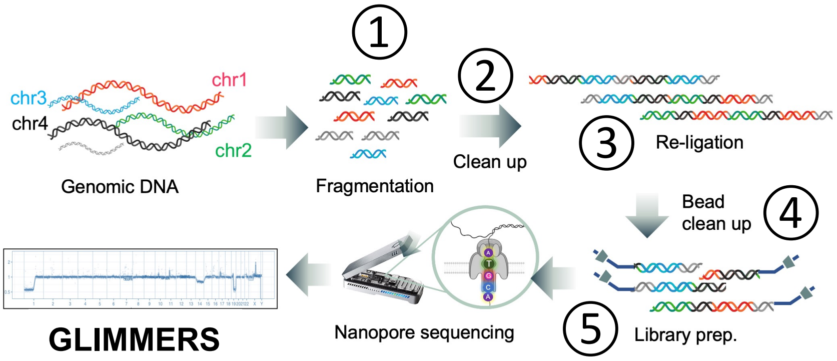

Checklist

- ❑ Anza 64 SaqAI (IVGN064-4)
- ❑ Anza T4 DNA Ligase Master Mix (IVGN210-4)
- ❑ Monarch® PCR & DNA Cleanup Kit (T1030S)
- ❑ Ampure XP bead (A63881) at RT
- ❑ Nuclease free water (B1500S)
- ❑ PCR tube, 1.5ml tube
- ❑ Magnetic Rack (0.2ml)
- ❑ PCR (37℃ & 30min; RT & 30min)
- ❑ Ethanol 200 proof

1. Restriction enzyme digestion

Thaw: Anza™ 64 SaqAI

- ❑ 1µg/17µl (NFW)
- ❑ 2µl Anza 10x Buffer
- ❑ 1µl Anza restriction enzyme

Mix reagents by pipetting
spin down

37℃ for 30 min

① cut-DNA, 20µl

SaqAI restriction site

5'...T|T A A...3'
3'...A A T|T...5'

2. Spin-column clean up

Prepare: Monarch® DNA Cleanup, label, tube & column

- ❑ 100µl DNA Cleanup Binding Buffer to 20µl cut-DNA
- ❑ Mix well by pipetting
- ❑ Insert column into collection tube and load sample onto column. Spin for 1 minute (~13,000 RPM), then discard flow-through.
- ❑ Re-insert column into collection tube. Add 200 µl DNA Wash Buffer and spin for 1 minute. Discarding flow-through. Repeat.
- ❑ Transfer column to a clean 1.5 ml microfuge tube.
- ❑ Add 12 µl of NFW to the center of the matrix. Wait for 1 minute, then spin for 1 minute to elute DNA.

② Cleaned-DNA, 10µl

3. Re-ligate

Anza T4 ligase

- ❑ 10µl Anza T4 DNA Ligase Master Mix to 500ng/10µl (NFW) cleaned-DNA
- ❑ Mix well by pipetting

RT for 30 min

③ Re-ligated DNA, 20µl

4. Bead clean-up

- ❑ 20µl Ampure beads to 20µl re-ligated DNA
- ❑ Mix well by flicking & spin down → RT for 5 min
- ❑ Place the PCR tube on a magnetic rack and wait for the supernatant to clear. Pipette off the supernatant and discard.
- ❑ Add 200µl of freshly prepared 80% ethanol and leave for 30 sec and remove the ethanol without disturbing the pellet. Repeat.
- ❑ Remove from magnet, spin down and replace on the magnet. Pipette off any residual supernatant and dry the beads for 1 min.
- ❑ Remove from magnet & re-suspend the beads in 7.5µl NFW. 2 min at RT.
- ❑ Place on magnet and wait till the elute is clear. Pipette out 7.5µl of elute which contains the ligated DNA.

④ Cleaned + ligated-DNA, 7.5µl

Rapid sequencing (RBK004)

- ❑ 2µl transposase to cleaned + ligated-DNA 7.5µl → 37℃ for 1.20 min, 80℃ for 1 min
- ❑ 0.8µl RAP → RT for 5 min
- ❑ 25µl SQB + 13µl LB + 10µl Adaptor-DNA + 2µl NFW
- ❑ 30 µl of thawed and mixed Flush Tether (FLT) to Flush Buffer (FLB) tube

⑤ Library prep

Reference: Exploiting nanopore sequencing for characterization and grading of IDH-mutant gliomas. Thidathip Wongsurawat *et al.* Brain Pathology, Volume34, Issue1, January 2024, e13203 https://doi.org/10.1111/bpa.13203
